# Partial nocturnal behaviour of giant mottled eels, *Anguilla marmorata*, in a small river, Amami‐Oshima Island, Japan, from acoustic telemetry

**DOI:** 10.1111/jfb.70492

**Published:** 2026-05-10

**Authors:** Hikaru Itakura, Ryoshiro Wakiya

**Affiliations:** ^1^ Atmosphere and Ocean Research Institute, University of Tokyo Kashiwa Chiba Japan; ^2^ Research and Development Initiative Chuo University Bunkyo‐ku Tokyo Japan; ^3^ Present address: Tokyo University of Marine Science and Technology, 4‐5‐7 Konan, Minato, Tokyo 108‐8477 Japan; ^4^ Present address: Atmosphere and Ocean Research Institute, University of Tokyo, Kashiwa, Chiba Japan

**Keywords:** acoustic telemetry, *Anguilla marmorata*, diel activity, home range

## Abstract

Anguillid eels typically exhibit nocturnal behaviour, but giant mottled eels *A. marmorata* are often observed during daytime in rivers on Amami‐Oshima Island, Japan. We investigated their diel activity patterns and spatial use with stationary‐receiver acoustic telemetry. Three tagged eels demonstrated partially nocturnal behaviour with some daytime activity. They displayed strong site fidelity to specific receiver locations, with occasional longer excursions to distant receiver locations. Because *A. marmorata* is a unique large‐bodied apex predator in tropical rivers and likely faces little predation risk, it might not need to restrict activity to night to avoid predators.

Fishes commonly display diel activity variations and spatial behaviours associated with feeding, resting, predator avoidance or reproduction. These behaviours are often influenced by changes in environmental and ecological conditions (Reebs, 2002). Understanding how fishes utilize their habitats in both time and space provides key insights into their ecological requirements and environmental constraints, thereby contributing to more effective conservation strategies. Observing diel behaviours is particularly relevant for catadromous freshwater eels of the genus *Anguilla*, because the nocturnal behaviour has been documented for some anguillids. Because of their commercial importance and population declines, studies using acoustic telemetry have focused on temperate anguillids, including American eels *A. rostrata* (Béguer‐Pon et al., 2015; Eissenhauer et al., 2026; Hedger et al., 2010), European eels *A. anguilla* (Ovidio et al., 2013; Walker et al., 2014), Japanese eels *A. japonica* (Itakura et al., 2018, 2022; Noda et al., 2021) and New Zealand eels *A. australis* and *A. dieffenbachii* (Jellyman & Sykes, 2003). These studies consistently showed that growth‐phase yellow eels are generally active at night and rest during the day. Nocturnal behaviours may not only help reduce predation risk but also enhance feeding efficiency as active predators (Glova & Jellyman, 2000; Reebs, 2002). In contrast, the riverine ecology of tropical anguillids, including their diel behavioural patterns, remains poorly understood.

Over more than a decade of field surveys on Amami‐Oshima Island, Japan, we have frequently observed the tropical anguillids, giant mottled eels *A. marmorata*, actively swimming during the daytime. The diel behaviour of temperate eels at least, is closely influenced by light intensity, which varies with habitat type and environmental conditions (Matsushige & Hibino, 2023; Reebs, 2002). For *A. japonica*, previous studies have shown that deep or turbid bottom environments are dark enough for eels to move even during the day, but they exclusively moved during night in shallow habitats (Itakura et al., 2018, 2022). The habitats of *A. marmorata* on Amami‐Oshima are mostly shallow and low in turbidity, but the species is frequently observed swimming during the day, which contradicts the pattern typically observed in temperate anguillid eels. Recent telemetry studies on *A. marmorata* conducted in the upper Cagayan River of the Philippines and in South Africa have also reported partially nocturnal behaviour, with individuals showing some daytime activity in deep and turbid habitats (Hanzen et al., 2021; Piper et al., 2022). In this study, we tracked *A. marmorata* using acoustic telemetry in a small river on Amami‐Oshima Island to examine their diel activity patterns and space use within freshwater habitat.

All experiments in this study, including fish sampling, handling and tracking, were conducted in accordance with the principles of ethics in animal experiments at the University of Tokyo.

This study was conducted in the Kominato‐Okawa River, a small river on Amami‐Oshima Island, Kagoshima prefecture, Japan (Figure S1). Nine acoustic receivers (AQRM‐1000, AquaSound Inc.; R1–R9) were deployed to the study area ranging 855–1440 m from the sea on 5 December 2015 by attaching the receivers to concrete blocks. The lowermost receiver (R9) was near the upper tidal limit, whereas a small weir was located 90 m upstream of the uppermost receiver (R1). Because the study area was basically shallow (<1 m) with riffles at and between receiver sites, detection ranges were expected to be limited. An acoustic range test near R3 confirmed that detections were rare beyond ~30 m. Accordingly, when a pulse from transmitters was detected, we inferred the eel to be within 30 m of that receiver.

Following receiver deployment, we captured three *A. marmorata* using electrofishing (LR‐20B, Smith‐Root, Inc., USA) near R3–R4 (Table 1). All fish (ID1–ID3; body length: 538, 580, 682 mm; body weight: 347, 457, 705 g) were confirmed as growth‐phase eels based on their body and pectoral fin colour (Hagihara et al., 2012). Under 10% eugenol anaesthesia solution (FA100, DS Pharma Animal Health Co., Ltd., Japan), we surgically implanted individually coded ultrasonic transmitters equipped with temperature‐depth sensors (AQPX‐1040PT, AquaSound Inc.; 43 mm length, 9.5 mm diameter, 1.8 g in water, 155 dB power output, 62.5 kHz frequency, 60 s delay interval, 90‐day battery life) following Itakura et al. (2018). The transmitters were implanted in the peritoneal cavity through a 20‐mm midline incision, which was then closed with two sutures. After confirming full recovery from anaesthesia based on the resumption of swimming in a recovery bucket, tagged fish were released at their capture sites on the same date of receiver deployment.

**TABLE 1:** Summary of the biological characteristics and tracking data of the tagged giant mottled eels (*Anguilla marmorata*) in the Kominato‐Okawa River, Amami‐Oshima Island, Japan.

ID	TL (mm)	BW (g)	Capture location	Duration tracked (days)	No. of detections	No. of detected movements	Percentage of movements (night)	Residency index	
								Day	Night
1	682	705	R3	77	7926	127	70.1%	0.4	0.6
2	580	457	R4	79	1370	183	63.9%	0.6	0.7
3	538	347	R4	79	958	404	54.0%	0.9	1

Abbreviations: BW, body weight; TL, total length.

Receivers were retrieved after 80 days on 23 February 2016 (10,254 detections; Table 1). All data processing and analyses were performed in R version 4.5.1 (R Core Team, 2025). Diel activity of eels is often inferred from the number of detections (detection frequency). Freshwater eels tend to shelter in refuges such as crevices and burrows in mud (Aoyama et al., 2005; Steendam et al., 2020), which may interrupt the transmission, causing fewer detections. Thus, previous studies have treated ‘detected’ as ‘active’ and ‘not detected’ as ‘inactive’ (Béguer‐Pon et al., 2015; Itakura et al., 2018; Jellyman & Unwin, 2017). In our dataset, however, detections were consistently recorded throughout the day (Figure S2), making that approach unsuitable. This is likely because (1) eels sheltering very close to a receiver may remain detectable even when inactive and (2) compared with some temperate eels (e.g., *A. japonica* inhabiting mainland Japan), the body of *A. marmorata* is often observed partially exposed while sheltering (Itakura et al. personal observation), reducing signal blockage. We therefore used movements between receivers as a proxy for activity. A movement was defined as consecutive detections at different receivers, and the linear distance between those receivers was considered as movement distance. The detected movements with intervals shorter than the delay interval of transmission (60 s) are indicative of the same ping being simultaneously detected by multiple receivers and were excluded (*n* = 51). Almost all these detections occurred between R3 and R5 (only 50‐m distance between receivers). This yielded 714 movement events. Day and night were delineated using sunrise/sunset times (from nearest town). The proportion of movements occurring during daytime or night‐time was calculated as the number of movements in that period divided by the total movement count. We also calculated a residency index as the number of days with at least one movement in the day (or night) divided by the detection window (total detection days from first‐to‐last detection for each eel) to evaluate diel activity (Noda et al., 2021; Villegas‐Ríos et al., 2013). During the study period, night‐time was on average 2 h 40 min longer than daytime. Therefore, if eel activity was strongly nocturnal, a substantially higher residency index during the night would be expected due to the longer duration of the night phase.

Of the detected movements, 62.7% ± 8.1% (mean ± SD) occurred at night, and the residency index tended to be higher at night than during the day, though differences were small (Table 1; Figure 1a). The smallest eel, ID3, moved the most and moved more during the day than the other eels (Table 1, Figures 1 and S3). Movement distances were also marginally greater at night than during the day, but the difference was not statistically significant (Figure S3). This indicates that the tagged eels exhibited partial nocturnal behaviour, moving to some extent during the day. Detections were concentrated near receivers R3–R4 where the eels were collected (ID1: 99.4%; ID2: 93.7%; ID3: 45.8%), indicating that eels primarily occupied those areas (Figure 1b). Occasionally, however, all eels made longer excursions up to R1 or to R8 followed by returning to R3–R4 (Figure 1b).

**FIGURE 1:**
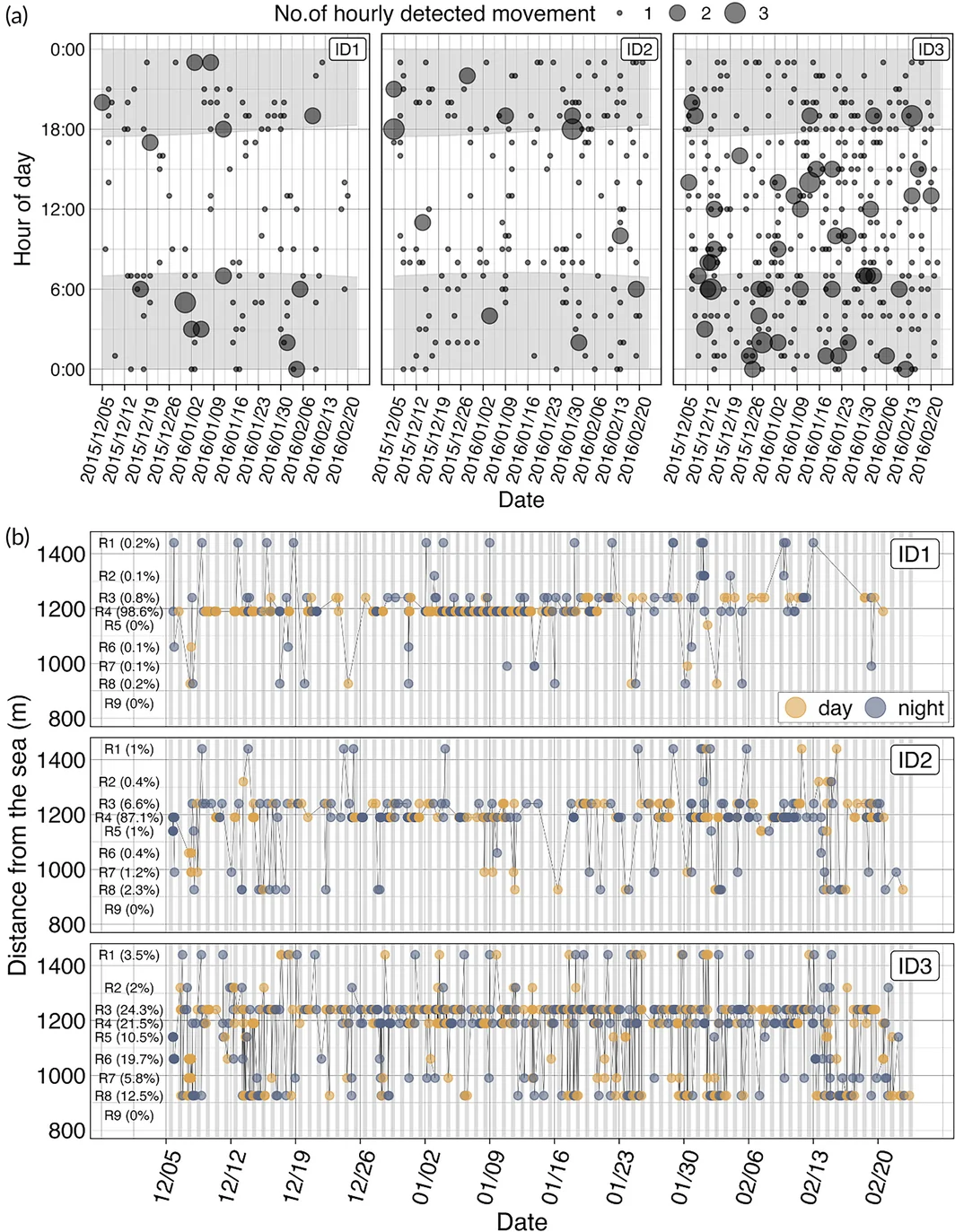
(a) Diel activity patterns of each tagged giant mottled eel, *Anguilla marmorata*, in the Kominato‐Okawa River, Amami‐Oshima Island, Japan. Circle sizes indicate numbers of hourly detections. (b) Changes in the detected receiver locations over time. R1–R9 indicate receiver identification numbers, and numbers in parentheses represent the proportion of receiver detections relative to total detections for each individual. Shading represents dark periods based on sunrise and sunset timings.

Because of the weir just upstream of R1 and no detections occurring at lowermost receiver R9, eels apparently remained within the study reach. However, only 41.6% of movements were between adjacent receivers, suggesting that eels could traverse a receiver's detection field in less than the 60‐s delay interval, by passing through small microhabitat side channels (e.g., bank crevices, boulder interstices, dense emergent riparian vegetation). The consistent returns to the R3–R4 area suggest that most activity remained there, with the maximum linear home range likely being <500 m (R1–R8 distance) or usually within about 100 m. Furthermore, because detections were often limited to a single receiver, the frequently used core area may have been even smaller (< 30 m), suggesting that they exhibit strong fidelity to specific sites, as reported for other anguillid eels (Itakura et al., 2018; Jellyman & Sykes, 2003; Ovidio et al., 2013; Verhelst et al., 2026). The mark–recapture experiment by Itakura and Wakiya (2020) similarly showed that most *A. marmorata* were recaptured at their past capture sites with an average linear home range <15 m.

Because only three individuals were tracked in this study, the results may not fully represent the movement behaviour of *A. marmorata*. However, movement studies on tropical anguillids remain extremely limited, and our observations provide preliminary insights into the diel behaviour and space use of the species in small tropical island rivers. We also acknowledge that the receiver proximity may make it difficult to distinguish true eel movements from instances where an eel was simply present within overlapping detection ranges without actual movement, particularly between receivers R3–R5 (interval: 50 m). Although a range test suggested detection ranges of <30 m, they likely vary depending on environmental conditions. Furthermore, although we excluded movements with intervals shorter than the delay interval of transmission as false movements, some movements may still reflect overlapping detections rather than actual movement.

The daytime activity of *A. marmorata* observed in this study is clearly different than the activity behaviour reported for temperate anguillid eels such as *A. japonica* (>98% of activity occurred at night; Itakura et al., 2022), *A. anguilla* (Bašić et al., 2019; Verhelst et al., 2017) and *A. rostrata* (Eissenhauer et al., 2026; Hedger et al., 2010). This daytime activity cannot be explained by low‐light conditions in deep or turbid habitats as reported for *A. japonica* (Itakura et al., 2018, 2022) and other fishes (Reebs, 2002). Therefore, this study and the daytime activity in previous studies (Hanzen et al., 2021; Piper et al., 2022) suggest that *A. marmorata* may typically be the apex predator in the tropical island habitats where it is present across the Indo‐Pacific. This would enable a partial nocturnal behaviour to be used in freshwater where there are no predators on medium‐to‐large eels such as we studied. The regular excursions observed in this study have also been reported for Japanese and American eels (Hedger et al., 2010; Noda et al., 2021), but one eel in the present study regularly moved even during daytime. It is known that diel activity and space use patterns of eels may vary with body size (Barry et al., 2016; Hanzen et al., 2021; Imbert et al., 2010; Wakiya et al., 2016). *A. marmorata* can grow to >1 m in total length on the study island (Wakiya et al., 2019), whereas the individuals tracked in this study were relatively small (<700 mm). Therefore, more eels, including both smaller and larger individuals, need to be studied to evaluate the movement behaviour of this remarkably widespread tropical anguillid eel. Such knowledge will inform the design and management of freshwater protected areas for this species as was proposed by Piper et al. (2022).

## AUTHOR CONTRIBUTIONS

H.I. and R.W. conceived the ideas, designed methodology and performed field surveys. H.I. analysed data and led writing of the manuscript. Both H.I. and R.W. contributed critically to the drafts and gave final approval for publication.

## FUNDING INFORMATION

This study was supported by the Environmental Research and Technology Development Fund by the Environmental Restoration and Conservation Agency.

## Supporting information


**Figure S1.** Locations of the Kominato‐Okawa River (length 9.4 km; catchment area 32.5 km^2^) on Amami‐Oshima Island, Japan, and the acoustic telemetry receivers deployed in the study river. In the lower panel, the circles indicate locations of each receiver and the numbers (and letter R) indicate receiver numbers. The mean ± SD distance between receivers was 73 ± 22 m (range 50–120 m). The lower panel was obtained from Google Earth imagery.
**Figure S2**. Diel activity patterns of each tagged giant mottled eel, *Anguilla marmorata*, in the Kominato‐Okawa River, Amami‐Oshima Island, Japan. The circle sizes indicate the number of hourly detections. Shading represents dark periods (i.e., night) based on sunrise and sunset timings. The eel ID1 likely remained near the receiver R4 (see Figure 1b) for several days in early January resulting in many detections.
**Figure S3**. Diel movement patterns of tagged giant mottled eels, *Anguilla marmorata*, in the Kominato‐Okawa River, Amami‐Oshima Island, Japan. (a) Distance travelled in each movement event. Shading represents dark periods (i.e., night) based on sunrise and sunset timings. (b) Comparison of each distance travelled between day and night. In the boxplots, the middle lines indicate the median, the boxes represent the 0.25 and 0.75 quartiles and the whiskers are the values that are within 1.5 of the interquartile range. Difference in movement distance between day and night was tested using a generalized linear mixed model (*glmer.nb* in the package *lme4* in R) with a negative binomial distribution and a log‐link function, which included each movement distance as a response variable; time of day (i.e., day or night) as a fixed effect; and individual as a random effect. The distances were not statistically different (estimate ± SE = 0.093 ± 0.056, *z* = 1.665, *p* > 0.05).

## Data Availability

The data that support the findings of this study are available from the corresponding author upon reasonable request.
