# Supplementary material for: Partial nocturnal behaviour of giant mottled eels, Anguilla marmorata, in a small river, Amami‐Oshima Island, Japan, from acoustic telemetry
Source: J Fish Biol. 2026 May 10;109(2):1307–11. doi: 10.1111/jfb.70492 (PMC13521694; doi:10.1111/jfb.70492)
Supplement: Supplementary file 1 [file JFB-109-1307-s001.docx]

**Supporting Information**

**Partial nocturnal behavior of Giant mottled eels, *Anguilla marmorata*, in a small river, Amami-Oshima Island, Japan, from acoustic telemetry**

Hikaru Itakura^1^, and Ryoshiro Wakiya^2^

^1^Atmosphere and Ocean Research Institute, University of Tokyo, Kashiwa, Chiba, Japan

^2^Research and Development Initiative, Chuo University, Bunkyo-ku, Tokyo, Japan

Current address

Hikaru Itakura (Corresponding Author): Tokyo University of Marine Science and Technology

Address: 4-5-7 Konan, Minato, Tokyo 108-8477 Japan

E-mail address: [itakura-useyaro@kaiyodai.ac.jp](mailto:itakura-useyaro@kaiyodai.ac.jp)

Ryoshiro Wakiya: Atmosphere and Ocean Research Institute, University of Tokyo, Kashiwa, Chiba, Japan.

**List of Contents**

1) Supplemental Figures

Figure S1 Locations of the study river and telemetry receivers.

Figure S2 Number of detections over time.

Figure S3 Movement distance over time and its difference between day and night.

**Figure S1** Locations of the Kominato-Okawa River (length 9.4 km; catchment area 32.5 km²) on Amami-Oshima Island, Japan, and the acoustic telemetry receivers deployed in the study river. In the lower panel, the circles indicate locations of each receiver, the numbers (and letter R) indicate receiver numbers. The mean ± SD distance between receivers was 73 ± 22 m (range 50–120 m). The lower panel was obtained from Google Earth imagery.

**Figure S2** Diel activity patterns of each tagged giant mottled eel, *Anguilla marmorata*, in the Kominato-Okawa River, Amami-Oshima Island, Japan. The circle sizes indicate the number of hourly detections. Shading represents dark periods (i.e., night) based on sunrise and sunset timings. The eel ID1 likely remained near the receiver R4 (see Fig. 1B) for several days in early January resulting in many detections.

**Figure S3** Diel movement patterns of tagged giant mottled eels, *Anguilla marmorata*, in the Kominato-Okawa River, Amami-Oshima Island, Japan. (a) Distance traveled in each movement event. Shading represents dark periods (i.e., night) based on sunrise and sunset timings; (b) Comparison of each distance traveled between day and night. In the boxplots, the middle lines indicate the median, the boxes represent the 0.25 and 0.75 quartiles, and the whiskers are the values that are within 1.5 of the interquartile range. Difference in movement distance between day and night was tested using a generalized linear mixed model (*glmer.nb* in the package *lme4* in R) with a negative binomial distribution and a log-link function, which included each movement distance as a response variable; time of day (i.e., day or night) as a fixed effect; and individual as a random effect. The distances were not statistically different (estimate ± SE = 0.093 ± 0.056, *z* = 1.665, *p* > 0.05)
